# Methods to advance health equity and social justice in healthcare: Protocol for a scoping review on the utilisation of routinely collected data

**DOI:** 10.1371/journal.pone.0306786

**Published:** 2024-07-10

**Authors:** Katie Chadd, Anna Caute, Anna Pettican, Pam Enderby

**Affiliations:** 1 School of Health and Social Care, University of Essex, Essex, United Kingdom; 2 Division of Public Health, University of Sheffield, Sheffield, United Kingdom; University of Victoria School of Nursing, CANADA

## Abstract

**Background:**

Many areas of healthcare are impacted by a paucity of research that is translatable to clinical practice. Research utilising real-world data, such as routinely collected patient data, may be one option to efficiently create evidence to inform practice and service delivery. Such studies are also valuable for exploring (in)equity of services and outcomes, and benefit from using non-selected samples representing the diversity of the populations served in the ‘real world’. This scoping review aims to identify and map the published research which utilises routinely collected clinical healthcare data. A secondary aim is to explore the extent to which this literature supports the pursuit of social justice in health, including health inequities and intersectional approaches.

**Method:**

This review utilises Arksey and O’Malley’s methodological framework for scoping reviews and draws on the recommended enhancements of this framework to promote a team-based and mixed methods approach. This includes searching electronic databases and screening papers based on a pre-specified inclusion and exclusion criteria. Data relevant to the research aims will be extracted from included papers, including the clinical/professional area of the topic, the source of data that was used, and whether it addresses elements of social justice. All screening and reviewing will be collaborative and iterative, drawing on strengths of the research team and responsive changes to challenges will be made. Quantitative data will be analysed descriptively, and conceptual content analysis will be utilised to understand qualitative data. These will be collectively synthesised in alignment to the research aims.

**Conclusion:**

Our findings will highlight the extent to which such research is being conducted and published, including gaps and make recommendations for future endeavours for real-world data studies. The findings from this scoping review will be relevant for practitioners and researchers, as well as health service managers, commissioners, and research funders.

## Introduction

Real-world data (RWD) is being increasingly recognised as fundamental to advancing many aspects of society, as our capacities for data collection and analysis grow exponentially. Healthcare is no exception to this, and many areas of healthcare industry are investing in, for example, developing wearables or apps to collect real-time, real-world data, and to answer pertinent clinical questions].

Routinely collected data [RCD) is health data often stored in electronic patient records, registries, administrative data or through monitoring apps/software, and is one form of RWD [1–4]. With some exceptions, traditionally, use of RCD has been disregarded as a source for research due to known vulnerabilities and potential biases, which are ‘off-set’ by presumed ‘rigorous’, highly controlled trials which have thus been long-considered the ‘gold standard’ of evidence [5]. Although, such studies are rarely conducted with those with complex and multiple conditions, or low incidence disorders. In response, in recent years the scientific community is starting to recognise RCD’s potential to offer an alternative and complementary source of data and evidence which can be used in addition and in adjunct to traditional research [6]. Distinct from observational studies—where strictly, data is still compared with control groups—RWD studies utilising RCD offer an alternative, descriptive methodology which can still utilise sophisticated statistical analyses and contribute meaningful evidence [7]. In recognition of RWD studies’ increasing acceptance in scholarly debate, the evidence produced by RWD studies is now ascribed its own typology–real-world evidence (or RWE). RWE is “typically derived from analyses of healthcare data outside of classical clinical trials” [p. 8] and thus describes the product of RWD research [7].

However, despite some increase in acknowledgement, advances in data collection, linkage and sharing, using RCD or RWD for research is seldom a selected approach, and when it is used it is often not labelled or recognised as such. Professionals and researchers are beginning to embrace RWD. For example, the Royal College of Speech and Language Therapists (RCSLT), the professional body for speech and language therapists in the UK, have made calls for other health professionals to embrace RWD and support practitioners to collate and analyse this, as they have done through their online tool and real world database [8].

The innate value of RCD in interrogating health care systems, processes, and outcomes–which are considerably challenging and unethical to examine through for example, randomised controlled trials—should not be dismissed [9]. A particularly valuable facet of RCD, through retrospective cohort studies and other designs, is the potential for exploring inequities in our health systems. This maybe among health services themselves (ie. distribution of resources across delivery functions) or indeed across populations which may be associated with locality, age, gender, ethnicity, socio-economic status and so on [10]. Only through understanding where inequities occur in our systems can we prioritise intervention to address gaps, that are evidence informed.

Addressing some of the flaws in much ‘traditional’ health research–RCD can include information from diverse caseloads that represent our multi-cultural, multi-ethnic, complex and diverse reality. This can mitigate the issue of under-representation of historically excluded populations within traditional trials’ participants [see 11 for an example in vaccine trials; 12 for psychiatry; and 13 for an example in cardiovascular health] [11–13]. Although this of course does still rely on data from those *already* ‘in the system’, which may mean some marginalised groups remain absent in the data (for example, those living without a fixed address, traveller communities and so on). However, where data can be harnessed, it does present is the potential for intersectional analyses [14] using RCD, i.e. using patient data to understand health and outcomes of individuals and/or groups of individuals with intersecting identities [see 15 for an example in mental health) [15], although this approach would rely on a comprehensive approach to data collection (for example, recording of patients’ ethnicity, religion, socio-economic status and so on).

Understanding more about the interactions between approaches to healthcare and characteristics of a person or population groups is imperative for problematizing inequities: how they present, where they present and what their manifestations, with regards to health status, are. Creating this knowledge is one step in creating change (to systems, to practices, to allocation of resources, for example), which in turn can support the pursuit of social justice. Social justice describes “full participation in society… resulting in equitable living and a just ordering of society” [16, p.955]. Should our knowledge (or evidence-base) about health be pinned upon the experiences and trajectories of a dominant socio-cultural-economic population group, inevitably social injustices and health inequities will occur for those whose identities diverge from it. Thus, creating evidence for healthcare that is not exclusionary or unequitable is a crucial step towards social justice.

Diversifying the direction of research can also be achieved through shifting the power in setting research agendas away from solely scholars, to a balanced perspective where the public (in health, this is typically service-users) and clinical practitioners have equal power in deciding priorities and undertaking research, which is pertinent to tackling social justice [17] Historically, research has not always been focused on areas that matter most to patients, or on procedures or interventions that are achievable by clinicians in practice. Creating such evidence is a costly and inefficient process, resulting in research and a waste of resources as well as stalling of innovations and improvements for patient care and outcomes [18]. Furthermore, there is often greater drive, investment, and interest in medical and nursing research, at detriment to the vital contribution of non-medical and non-nursing health fields (such as the allied health professions–which include physiotherapists, occupational therapists, speech and language therapists among others–as well as psychologists and biomedical professionals). With data at these professionals’ fingertips, RCD research presents an opportunity for front-line clinicians to ask and answer pertinent clinical questions that can instantaneously influence their practice. This is particularly valuable for health care professionals whereby ‘traditional’ research is lacking.

Thus, using RCD to create real-world evidence is one avenue for increasing diversity in the evidence that we create with greater potential to directly impact practice, thus facilitating equitable and just clinical practices and service delivery [10].

Given recent and numerous policy directives to enhance equality and equitability of health and wellbeing services across the UK, and globally–especially since COVID-19- it is timely to revisit the RCD and RWD agenda, the landscape of the research using it, and how it can be exploited for the pursuit of social justice.

The objectives of the review are to:

1. Document the use of routinely collected data research in non-medical and non-nursing health fields in the published peer-reviewed literature
2. Describe the researcher team (clinician or academic), research questions, data sources and methods used in these studies
3. Evaluate the extent to which routinely collected data research has aimed to address issues pertaining to social justice, specifically to document reference to:
  - tackling issues of health inequity or inequality
  - inclusion of typically underserved populations in their samples
  - whether it has incorporated an intersectional analysis
4. Use the findings to create recommendations for future real-world data studies to advance the evidence base in non-medical and non-nursing health fields, and particularly further the pursuit of social justice in health.

The research question is: What research has been published in non-medical and non-nursing health fields that utilises routinely collected data as its primary method, and to what extent does it address key issues pertaining to social justice?

## Methods

A scoping review will be undertaken. A scoping review is appropriate to address the research objectives and answer the questions which are exploratory in nature and strive to determine the ‘coverage’ of the body of literature on routinely-collected data in non-medical and non-nursing health [19]. This scoping review will follow the 6-stage framework for scoping reviews as set out by Arksey and O’Malley [20] and include additional steps that follow the enhancements of this framework as suggested by Westphal et al. [21]. In order to develop and complete the protocol, some of these steps have been completed prior to publication of this protocol. The completed and intended stages are outlined below.

### Stage 1) Specify the research question

In line with the scoping review framework, a clearly defined research question is required [20], which can be further supported by use of an identified framework [21]. To further inform on the conceptualisation of our research question, an informal rapid scan of relevant literature was undertaken. This review indicated that the expanse of literature relating to real-world data was vast and across many disciplines and health sectors, and to a large extent appeared to be centred on med tech and drug development. For example, a rapid review published by Chen et al. (2021) provided an overview of ‘drug development studies’ that leveraged real-world data, published up until July 2020 which included 16 studies [22]. As we were particularly scoping research conducted outside the traditional paradigms of clinical trials, this indicated the need to clearly define these concepts within our question and review methods. This prompted specification of ‘routinely collected data’ over the more generic ‘real world data’ which reflected our narrower focus. Since this scoping review is predominantly a *methodology review*, but also focused on qualitative aspects of studies such as their research aims in relation to social justice, the Sample, Phenomenon of Interest, Design, Evaluation, Research type (‘SPIDER’) framework was chosen to guide our question development. The SPIDER framework has been discussed as an approach for mixed-methods research and for guiding systematic reviews [23,24]. Below, we outline the SPIDER concepts and the research question.

- **Sample:** Peer-reviewed studies that have used routinely collected data in non-medial, non-nursing health fields
- **Phenomenon of Interest:** How routinely collected data has been used, by whom, and whether it addresses issues related to social justice (health inequities, inequalities and inclusive sampling)
- **Design:** Studies using routinely collected data *outside* of traditional clinical design paradigms
- **Evaluation:** The volume of research published and the extent to which it addresses our phenomena of interest
- **Research type:** A scoping review

Thus, our research question is: *“What research has been published in the non-medical and non-nursing health field that utilises routinely collected data as its primary method*, *and to what extent does it address key issues pertaining to social justice*?

### Stage 2) Identify the relevant literature

The second stage of the scoping review should focus on identifying the literature [20], which can be enhanced by using a systematic team-based approach [21]. Table 1 below outlines the team that was assembled who aided in the development of the scoping review protocol including identification of relevant literature (Table 1).

**Table 1:** Matrix of expertise of scoping review team members.

Expertise		Team			
		AP	AC	KC	PE
Systematic literature reviewing					x
Real world data / routine data		x	x	x	x
Health area	Occupational therapy	x			x
	Speech and language therapy		x	x	x
Expert consultant					x

#### Search strategy and terms

Initial search terms and search string was developed for the databases selected. Table 2 outlines the key concepts, search terms/phrases and operators identified. Health fields to be searched have been limited specifically to the eight largest non-medical, non-nursing regulated health professions in the United Kingdom which comprise 80% of the non-medical and non-nursing health workforce [25], to ensure the scoping review maintains feasibility (*physiotherapists*, *occupational therapists*, *radiographers*, *paramedics*, *practitioner psychologists*, *biomedical scientists and speech and language therapists*) (Table 2). As a recommended enhancement to the Arksey and O’Malley process, Westphaln et al also suggest inviting a librarian to help guide the search thus a health care librarian and information specialist was consulted [21]. A ‘NOT’ operator for the search term ‘survey’ at the conservative level of Title was applied, following trial searches which yielded an unprecedented number of survey-based studies (often reporting as service evaluations or audits) which did not use RCD, to manage the feasibility of the scoping exercise. Three health and medical electronic databases of interest have been identified for use in this scoping review due to their relevance to the topic. These are detailed and justified below:

1. **CINAHL Ultimate:** this database is the primary index for allied health literature, as allied health forms a primary area of interest CINAHL Ultimate is a crucial database to include.
2. **MEDLINE Ultimate:** this is database for medical and related professions literature and full text articles for 1650 journals.
3. **PubMed:** As the digital archive for the National Library of Medicine (United States), PubMed is another large database for biomedical literature, searching this database will support identification of relevant literature.

**Table 2:** Draft review concepts with search terms/phrases, levels, operators and limiters.

Concepts	1	AND	2	NOT	3	NOT	3
	Routine data / real-world data		Health area		Other study types		Other methods (non-routine)
Level	Title		Abstract		Title		Abstract
Terms	“Routine data” OR “routine clinical data” OR “routinely collected” OR “routine clinical data” OR “real world data” OR “real world evidence” OR “electronic health” OR “medical records” OR “health record” OR “patient record” OR “patient data” OR (“registry” NOT "trials registry") OR “service data” OR “service evaluation” OR “audit” OR “case note” OR “case notes”		((speech OR language OR occupational OR physical OR physio) AND (therap* OR patholog*)) OR physiotherap* OR psycholog* OR radiography OR Radiographer* OR paramedic* OR “biomedical scientist”		"systematic review" OR meta* OR trial OR survey		Interview* OR “focus group” OR “focus groups”
Limiters							
	Academic journals In English						

**Fig 1:**
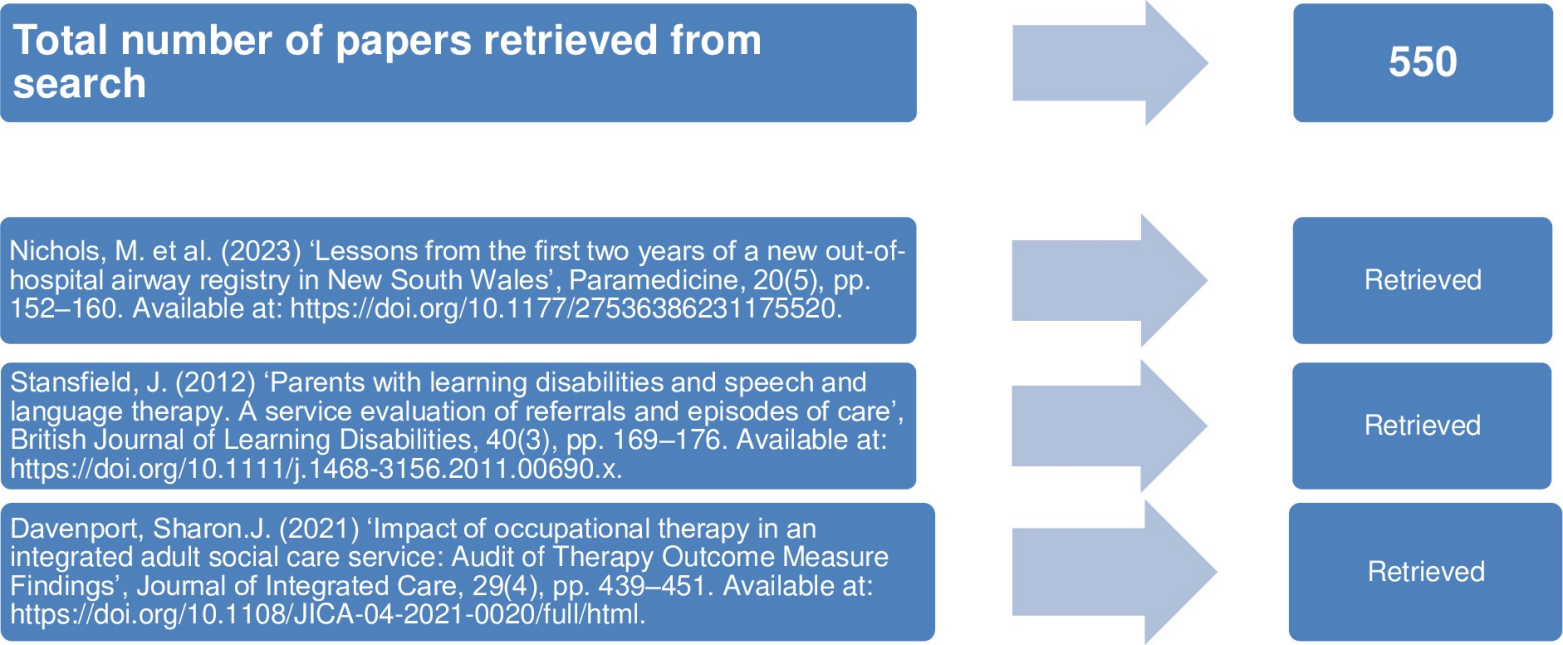
Target articles and results from draft CINAHL Ultimate search.

The search strategy was trialled in the CINAHL Ultimate database by ascertaining whether it captured three target articles known to the research team that would be expected to be obtained and included in the review, through combining the given search in addition to the first author’s name at the level of Author, for each target article. (Fig 1). The draft search also served as a feasibility test for the review, which was ascertained by a retrieval of <1000 articles.

The search strategy will be iteratively developed and adapted using relevant basic operators to the electronic databases searched. There will be no date filters applied for this search to ensure wide coverage and will run up until current of the date of the search (planned January 2024). Once finalised, the search strategy will be fully reported using the PRISMA Extension for Scoping Reviews [26].

### Stage 3) Select studies

Selecting studies for the scoping review is the next stage, which, although iterative, should be transparent and reproducible in line with the scoping review frameworks used in this project [20,21]. Articles retrieved from the database searches will be imported and stored in Rayyan software. This will facilitate simple deduplication and support the subsequent screening of Titles and Abstracts for inclusion or exclusion. The criteria for article screening are reported in Table 3, though due to the iterative nature these may refined when the screening takes place (Table 3).

**Table 3:** Inclusion and exclusion criteria for article screening.

Include	Exclude
Peer-reviewed journal articles	Studies using non-routine data: studies using specialised apps or tracking software to collate data that would not otherwise typically be collected in clinical services OR service evaluations using surveys or interviews OR survey studies OR observational studies.
Study designs which clearly indicate use of routine clinical (patient) data so long as the studies are exploring clinical or service-related questions and are answered	Trials exploring the development/ feasibility/accuracy/effectiveness/adoption/ implementation of new data collection tools (such as new electronic health record systems or apps) UNLESS a clinical question was also addressed which used the data that was collected
Written in English or translated to English.	Studies exploring perspectives on collecting patient data, using routine-data for research or similar
Studies that primarily concern the selected healthcare professions, or that include explicit recommendations for the selected professions, or that relate to broad disciplinary areas where the healthcare professions are known to hold a considerable role (for example, ‘stroke rehabilitation’ or cerebral palsy).	Study protocols
	Review or discussion papers
	Literature reviews, including systematic reviews and meta-analyses
	Commentaries
	Conference proceedings
	Non peer-reviewed articles
	Written in languages other than English with no translated version available

Two members of the research team will pilot the study screening protocol on the same random 5% of papers (and agreement will be calculated). Disagreements will be indicated through Rayyan, and be reconciled through conversation, and points underpinning the reconciliation will be used to refine the inclusion and exclusion criteria further. Once refined, the remainder of the papers will be screened for inclusion and exclusion by 2 members of the team, which will be compared. A third member will screen any studies where decision-making was either a) not possible based on the title or abstract (thus marked as ‘Maybe’ on the software) or b) inconsistent between reviewers, as indicated by the software. If a third reviewer is still unable to determine inclusion or exclusion, the full text of these studies will be reviewed. An inter-rater reliability co-efficient will be calculated for the study screening process.

Each remaining study will then undergo full-text screening by researcher KC and at least one other member of the team. Studies will be assigned to team members which match their knowledge or expertise as much as possible (for example, AP will serve as a second screener for full-text articles related to occupational therapy). Again, a third research team member will serve as an arbitrator for resolving disagreements, which will be indicated through Rayyan. A final list of include papers will be presented to the whole research team for verification and agreement. All decision-making will be documented, and an inter-rater reliability co-efficient will also be calculated for this part of the process. The study selection process will be fully reported using the PRISMA Extension for Scoping Reviews [26].

### Stage 4) Extracting, mapping, and charting the data

All identified papers will be imported into Rayyan which is an AI-driven software that facilitates collaborative reviews by providing a platform to compile all papers and support screening and extraction processes. Descriptive data regarding the studies will be extracted, and the ‘Reporting of studies Conducted using Observational Routinely-collected health Data (RECORD)’ statement [27] will be used as a framework to identify additional key elements for data relevant to the review (ie. rationale, objective, setting, participants, variables, data sources). Constructs related to social justice will also be extracted. Table 4 outlines the draft extraction framework, which includes qualitative and quantitative aspects (Table 4). This mixed methods approach is advantageous in that it will help answer our research questions more richly, provide more evidence and provide a way to corroborate findings [21]. The process may evolve after the extraction stage has initiated, reflecting an iterative process. When the PI has extracted data from 5% of the papers, a preliminary analysis will be done and the extraction framework will be revised and adapted as necessary in consultation with the group [20].

**Table 4:** Draft data extraction framework.

**1. Descriptive data**	Authors	
	Lead author institution (academic / clinical / mixed)	
	Research team composition (academic institutions, clinical institutions)	
	Date of publication	
	Journal of publication	
	Country (of first author)	
	Related health profession or clinical area	
	Number of datasets explored	
	Aim or background references translation of evidence to practice, implementation of research, research to practice gap.	
**2. RECORD statement**	Rationale	
	Objective	
	Setting	
	Participants	
	Variables	
	Data sources	
	Quantitative or Qualitative or Mixed	
**3. Social justice**	Social justice^1^	Aim: central / secondary / mention
		Nature of link to social justice
	Health inequities	Aim: central / secondary / mention
		Nature of link to health inequities
	Under-represented groups^3^	Aim: central / secondary / mention
		‘Seldom heard’ population/s included
	Intersectionality^4^	Aim: central / secondary / mention
		Nature: Intersecting variables examined and method

^1^ For the purposes of this review, when classifying studies as whether they address social justice centrally or not, we will use the following descriptor: The study acknowledges and purposively addresses issues related to the broad paradigm of ‘equal rights’. In health care research this may include (but is not limited to), topics relating to: Distribution of and access to healthcare, opportunities to benefit from health care, and particularly these in relation to marginalised communities. Standard intervention /comparison studies are excluded from this unless they acknowledge and address issues pertaining to inequity, inequality and marginalised communities. This could also be a secondary aim.

^2^For the purposes of this review, when classifying studies as whether they address health inequities (or inequalities) as a central aim or not, we will use the following descriptor: The study acknowledges and purposively addresses health related process or outcomes that are evidenced, or seek to be evidenced, as inequitable across society or the empirical context. Standard intervention /comparison studies are excluded from this unless they acknowledge and address issues pertaining to distinctive inequalities between marginalised communities. This may be a secondary aim.

^3^For the purposes of this review, when classifying studies as whether they include under-represented groups as a central aim or not, we will use the following descriptor: The study has a specific aim to amplify the experience/voice etc of, collects data from and specifically describes characteristics (and corresponding results) of individuals or groups that are typically associated with marginalisation as defined NHS CORE20PLUS ‘population groups’ and ‘inclusion groups’ (people with a learning disability and autistic people; coastal communities with pockets of deprivation hidden amongst relative affluence; people with multi-morbidities; young carers, looked after children/care leavers and those in contact with the justice system, other groups that share protected characteristics as defined by the Equality Act 2010 (i.e. groups of people with a shared ethnicity/race, gender identity, sexual orientation and so on); people experiencing homelessness, drug and alcohol dependence, vulnerable migrants, Gypsy, Roma and Traveller communities, sex workers, people in contact with the justice system, victims of modern slavery and other socially excluded groups). This framework has been selected as a policy-informed approach which is comprehensive and holistic in its inclusion of any group that share one or more (intersecting) protected characteristics as well as other specific groups know to be vulnerable to health inequality.

^4^For the purpose of this review, when classifying studies as whether they address intersectionality, we will use the following descriptor: The study acknowledges and purposively addresses the topic of intersectionality, intersecting identities or concurrent identity labels which may reinforce inequity and marginalistion. In health care research this may include (but is not limited to) an analysis of interaction effects between population groups with 2 or more intersecting variables. For example, looking at different in patient outcomes which may be broken down by ethnicity *and* socio-economic background *and* gender (such as comparing the most socio-economically disadvantaged Black male patients and most socio-economically disadvantaged White male patients).

The data extraction and charting process will be enhanced by being piloted by 2 members of the team on 5 randomly selected papers each, with updates made to charting templates and variables, as required, before conducting the remainder of the review [21]. After the refining of the charting process, the PI will independently chart 100% of the included papers. A second team member/s will chart a random sample of 30% of the included papers and third member will address questions or resolve discrepancies. Should consistent discrepancies arise, the PI may need to revisit papers already charted to correct for tweaks/changes to the charting process, that may arise from resolving conflicts.

### Stage 5) Summarise, synthesize and report the results

Summary and synthesis from will be led by KC in consultation with the research team. Descriptive statistics will be used to summarise key quantitative general characteristics of the papers. The qualitative data components (such as how the paper refers to research-to-practice gaps, or the nature of the article’s link to health inequities) will be analysed at the level of the phrasing reported within the extraction process which will be imported and analysed using NVivo software. A conceptual content analysis approach will be utilised, which will involve inductively developing codes for each of the concepts reported and creating a coding framework. The presence of codes will be quantified, and illustrative quotations relevant to each code provided. These data will be synthesised together and in alignment with the research questions, aims and objectives.

### Stage 6) Integrate expert consultation

The wider research team involves senior researchers who are consulting on this project and their views on the analysis and a summary of the findings will be sought in the first instance. A wider consultation on the draft protocol for the scoping review took place between 2 November 2023 and 07 December 2023 through submitting a draft on the Open Science Framework platform and sharing this through social media and institutional research networks. In this period, one set of comments were received by the PI which resulted in the addition of the ‘*intersectionality’* component of the scoping review.

Furthermore, a similar exercise regarding the summary of the draft findings will take place online, through publication of the draft findings on a shared platform with an open invitation for feedback and comments. Specifically, health professional bodies will be approached and asked to provide feedback both from the organisation and the membership. The feedback elicited during the consultation period will be used to frame the findings in the most relevant, suitable, and meaningful way. The final scoping review and its findings will be reported in a peer-reviewed academic journal and disseminated at conferences as appropriate.

## Discussion

We anticipate that through conducting this research, practices of routinely-collected data research will be unveiled which will open the opportunity for critical analysis of such real-world data research in the larger health research field. In so doing, we aim to contribute to the ongoing discussions around what real-world data studies can (and do) offer, particularly in non-medical and non-nursing health. Moreover, the close examination of whether, and how, these studies orient to issues pertaining to social justice will offer novel insights into whether current studies are exploiting the potential of routinely-collected data studies to address such questions.

## Supporting information

S1 Checklist PRISMA-P (Preferred Reporting Items for Systematic review and Meta-Analysis Protocols) 2015 checklist: Recommended items to address in a systematic review protocol*. (DOC)
