# Supplementary material for: Methods to advance health equity and social justice in healthcare: Protocol for a scoping review on the utilisation of routinely collected data
Source: PLoS One. 2024 Jul 10;19(7):e0306786. doi: 10.1371/journal.pone.0306786 (PMC11236175; doi:10.1371/journal.pone.0306786)
Supplement: S1 Checklist — (DOC) [file pone.0306786.s001.doc]

**PRISMA-P (Preferred Reporting Items for Systematic review and Meta-Analysis Protocols) 2015 checklist: recommended items to address in a systematic review protocol***

| Section and topic | Item No | Checklist item | Author comments |
| --- | --- | --- | --- |
| ADMINISTRATIVE INFORMATION | | |  |
| Title: |  |  |  |
| Identification | 1a | Identify the report as a protocol of a systematic review | 1a. This is indicated in the title, abstract and throughout the paper.  1b. n/a |
| Update | 1b | If the protocol is for an update of a previous systematic review, identify as such |  |
| Registration | 2 | If registered, provide the name of the registry (such as PROSPERO) and registration number | 2. This is a scoping review not a systematic review therefore is not registered on PROSPERO. |
| Authors: |  |  |  |
| Contact | 3a | Provide name, institutional affiliation, e-mail address of all protocol authors; provide physical mailing address of corresponding author | 3a. Name, institution and contact information of authors is provided.  3b. Contributions to the protocol from authors have been provided. |
| Contributions | 3b | Describe contributions of protocol authors and identify the guarantor of the review |  |
| Amendments | 4 | If the protocol represents an amendment of a previously completed or published protocol, identify as such and list changes; otherwise, state plan for documenting important protocol amendments | 4. n/a |
| Support: |  |  |  |
| Sources | 5a | Indicate sources of financial or other support for the review |  |
| Sponsor | 5b | Provide name for the review funder and/or sponsor | 5a. No sources are to be declared – this is indicated on the manuscript.  5b. No funding was received - this is indicated on the manuscript.  5c. N/a |
| Role of sponsor or funder | 5c | Describe roles of funder(s), sponsor(s), and/or institution(s), if any, in developing the protocol |  |
| INTRODUCTION | | |  |
| Rationale | 6 | Describe the rationale for the review in the context of what is already known | 6. The rationale is provided in the Introduction section, including an overview of what is already known. |
| Objectives | 7 | Provide an explicit statement of the question(s) the review will address with reference to participants, interventions, comparators, and outcomes (PICO) | 7. The research question is clearly stated. As this is a scoping review not a systematic review, PICO is not required. However, we have utilised the SPIDER question framework and have referenced this as such. |
| METHODS | | |  |
| Eligibility criteria | 8 | Specify the study characteristics (such as PICO, study design, setting, time frame) and report characteristics (such as years considered, language, publication status) to be used as criteria for eligibility for the review | 8. the study characteristics to be considered for inclusion and exclusion are provided in the Methods section – Stage 3 ‘Select studies’. |
| Information sources | 9 | Describe all intended information sources (such as electronic databases, contact with study authors, trial registers or other grey literature sources) with planned dates of coverage | 9. All intended information sources and planned dates of coverage are outlined in the Methods section – Stage 2 ‘identify the relevant literature’. |
| Search strategy | 10 | Present draft of search strategy to be used for at least one electronic database, including planned limits, such that it could be repeated | 10. Draft search strategy is provided clearly in Table 2. |
| Study records: |  |  |  |
| Data management | 11a | Describe the mechanism(s) that will be used to manage records and data throughout the review | 11a. Detail on the mechanism to manage records is given in Methods – Stage 3 ‘Select studies’ and Stage 4 ‘Extracting, mapping and charting the data |
| Selection process | 11b | State the process that will be used for selecting studies (such as two independent reviewers) through each phase of the review (that is, screening, eligibility and inclusion in meta-analysis) | 11b. The process for selecting studies is reporting in Methods – Stage 3 ‘Select studies’ including the number of reviewers at each stage of the review. |
| Data collection process | 11c | Describe planned method of extracting data from reports (such as piloting forms, done independently, in duplicate), any processes for obtaining and confirming data from investigators | 11c. The process for extraction in terms of piloting, numbers of reviewers and reconciliation is outlined in Methods – Stage 4 ‘Extracting, mapping and charting the data’ |
| Data items | 12 | List and define all variables for which data will be sought (such as PICO items, funding sources), any pre-planned data assumptions and simplifications | 12. A draft extraction framework is provided in Table 4 which lists and defines all variable for which data will be sought. |
| Outcomes and prioritization | 13 | List and define all outcomes for which data will be sought, including prioritization of main and additional outcomes, with rationale | 12. As a scoping review rather than systematic review, we do not have defined outcomes other than what is collected in the data extraction (as above). |
| Risk of bias in individual studies | 14 | Describe anticipated methods for assessing risk of bias of individual studies, including whether this will be done at the outcome or study level, or both; state how this information will be used in data synthesis | 12. As a scoping review rather than systematic review, we will not be examining risk of bias. |
| Data synthesis | 15a | Describe criteria under which study data will be quantitatively synthesised | 15a. The process of synthesis of data is outlined in Methods – Stage 5 ‘Summarise, synthesize and report the results’. This includes how descriptive statistics will be used to quantitatively synthesise relevant components. |
|  | 15b | If data are appropriate for quantitative synthesis, describe planned summary measures, methods of handling data and methods of combining data from studies, including any planned exploration of consistency (such as I^2^, Kendall’s τ) | 15b. The process of synthesis of data is outlined in Methods – Stage 5 ‘Summarise, synthesize and report the results’. As a scoping review, rather than a systematic review, summary measures and data combining will not be performed. |
|  | 15c | Describe any proposed additional analyses (such as sensitivity or subgroup analyses, meta-regression) | 15c. As a scoping review, rather than a systematic review, analyses such as these will not be performed. |
|  | 15d | If quantitative synthesis is not appropriate, describe the type of summary planned | 15d. The process of synthesis of data is outlined in Methods – Stage 5 ‘Summarise, synthesize and report the results’. This includes how qualitative data will be analysed and summarised. |
| Meta-bias(es) | 16 | Specify any planned assessment of meta-bias(es) (such as publication bias across studies, selective reporting within studies) | 16. As a scooping review rather than a systematic review, this will not be examined. |
| Confidence in cumulative evidence | 17 | Describe how the strength of the body of evidence will be assessed (such as GRADE) | 17. As a scooping review rather than a systematic review, this will not be examined. |

*** It is strongly recommended that this checklist be read in conjunction with the PRISMA-P Explanation and Elaboration (cite when available) for important clarification on the items. Amendments to a review protocol should be tracked and dated. The copyright for PRISMA-P (including checklist) is held by the PRISMA-P Group and is distributed under a Creative Commons Attribution Licence 4.0.**

*From: Shamseer L, Moher D, Clarke M, Ghersi D, Liberati A, Petticrew M, Shekelle P, Stewart L, PRISMA-P Group. Preferred reporting items for systematic review and meta-analysis protocols (PRISMA-P) 2015: elaboration and explanation. BMJ. 2015 Jan 2;349(jan02 1):g7647.*
